# Downregulation of pro-surfactant protein B contributes to the recurrence of early-stage non-small cell lung cancer by activating PGK1-mediated Akt signaling

**DOI:** 10.1186/s40164-023-00455-6

**Published:** 2023-11-09

**Authors:** Hao Luo, Qing Li, Ren-Tao Wang, Liang Zhang, Wei Zhang, Meng-Sheng Deng, Yuan-Yuan Luo, Xintong Ji, Yongheng Wen, Xuan-Rui Zhou, Bo Xu, Dong Wang, Bin Hu, Hua Jin, Cheng-Xiong Xu

**Affiliations:** 1 School of Medicine, Chongqing University, Chongqing, 400030 China; 2 Cancer Center, Daping Hospital, Army Medical University, Chongqing, 400042 China; 3 College of Pulmonary and Critical Care Medicine, Chinese PLA General Hospital, Beijing, China; 4 Department of Thoracic Surgery, Daping Hospital, Army Medical University, Chongqing, 400042 China; 5 Sichuan Cancer Hospital & Institute, Sichuan Cancer Center, University of Electronic Science and Technology of China, Chengdu, 610041 China; 6 State Key Laboratory of Trauma Burn and Combined Injury, Daping Hospital, Army Medical University, Chongqing, 400042 China; 7 Chongqing Key Laboratory of Intelligent Oncology for Breast Cancer, Chongqing University Cancer Hospital and Chongqing University School of Medicine, Chongqing, 400030 China

**Keywords:** Recurrence, Early-stage NSCLC, Pro-SFTPB, PGK1, Akt pathway

## Abstract

Recurrence is one of the main causes of treatment failure in early-stage non-small cell lung cancer (NSCLC). However, there are no predictors of the recurrence of early-stage NSCLC, and the molecular mechanism of its recurrence is not clear. In this study, we used clinical sample analysis to demonstrate that low levels of expression of precursor surfactant protein B (pro-SFTPB) in primary NSCLC tissue compared to their adjacent tissues are closely correlated with recurrence and poor prognosis in early-stage NSCLC patients. In vitro and in vivo experiments showed that downregulation of pro-SFTPB expression activates the Akt pathway by upregulating PGK1, which promotes metastasis and tumorigenicity in NSCLC cells. We then demonstrated that pro-SFTPB suppresses the formation of the ADRM1/hRpn2/UCH37 complex by binding to ADRM1, which inhibits PGK1 deubiquitination, thus accelerating ubiquitin-mediated PGK1 degradation. In summary, our findings indicate that low expression of pro-SFTPB in primary NSCLC compared to their adjacent tissue has potential as a predictor of recurrence and poor prognosis in early-stage NSCLC. Mechanistically, downregulation of pro-SFTPB attenuates inhibition of ADRM1-deubiquitinated PGK1, resulting in elevated levels of PGK1 protein; this activates the Akt pathway, ultimately leading to the progression of early-stage NSCLC.

**Supplementary Information:**

The online version contains supplementary material available at 10.1186/s40164-023-00455-6.

## Background

Lung cancer is the leading cause of cancer incidence and mortality worldwide [1]. Non-small cell lung cancer (NSCLC) is the main subtype of lung cancer, accounting for approximately 85% of all lung cancers [2]. Although various small molecule drugs and targeted therapies have been developed in recent years for NSCLC, research has mainly focused on advanced NSCLC [3–5]. Diagnosis at early stages is vital for improving the survival rate of NSCLC patients, and surgery is the primary treatment for early-stage NSCLC [6–8]. However, approximately 30–55% of patients with early-stage NSCLC develop recurrence after surgery and die of the disease [6–8]. Unfortunately, there are currently no biomarkers that can predict the recurrence of early-stage NSCLC, and the factors and molecular mechanisms that promote the recurrence of early-stage NSCLC are still largely unknown.

Surfactant protein B (SFTPB), a major component of pulmonary surfactant, is strictly required for breathing, and its absence is associated with lethal respiratory failure in humans [9]. Interestingly, recent studies have shown that both mature SFTPB and its precursor have physiological functions. In particular, abnormal expression of the precursor of SFTPB (pro-SFTPB) may be associated with NSCLC development and progression. However, findings in this area are inconsistent. For example, some studies have shown that high expression of pro-SFTPB is a predictor of lung cancer [10] and that it correlates with lymph node metastasis in NSCLC [11]. However, other studies reported that SFTPB is downregulated in lung adenocarcinoma patients with relapse compared to those without relapse [12], and that low expression of surfactant proteins correlates with a lower overall survival rate in lung adenocarcinoma patients with brain metastasis [13]. Interestingly, a recent report showed that both nondetectable status and high levels of plasma pro-SFTPB are significantly associated with lung cancer risk [14]. These findings suggest that both abnormal underexpression and abnormal overexpression of pro-SFTPB may be involved in promoting lung cancer progression. In particular, low expression of pro-SFTPB may be related to the metastasis and recurrence of lung cancer, but further confirmation is needed, and the molecular mechanism of this effect is still unclear.

In this study, we used clinical samples to demonstrate that low expression of pro-SFTPB in primary NSCLC, compared to its expression in adjacent tissue, is strongly associated with recurrence in early-stage NSCLC. Early-stage NSCLC patients with low levels of serum pro-SFTPB were also found to have shorter recurrence-free survival and shorter overall survival than healthy individuals. In addition, in vitro and animal experiments showed that downregulation of pro-SFTPB significantly enhances the metastatic ability and tumorigenicity of NSCLC cells. Mechanistically, downregulation of pro-SFTPB caused upregulation of phosphoglycerate kinase 1 (PGK1), which led to activation of the Akt signaling pathway, thereby stimulating NSCLC progression. Furthermore, we demonstrated that pro-SFTPB negatively regulates PGK1 protein levels by binding to ADRM1 and suppressing complex formation by ADRM1, hRpn2 and UCH37; this weakened ADRM1/hRpn2/UCH37 complex-mediated PGK1 deubiquitination, thereby facilitating degradation of the PGK1 protein in NSCLC. In conclusion, low expression of pro-SFTPB in primary tumors compared to adjacent tissue is a potentially robust biomarker for recurrence of early-stage NSCLC, and downregulation of pro-SFTPB expression promotes early-stage NSCLC recurrence by activating the PGK1-mediated Akt signaling pathway.

## Methods

### Reagents

Cycloheximide, MG132, 2-(4-Amidinophenyl)-6-indolecarbamidine dihydrochloride (DAPI), Dulbecco`s Modified Eagle Medium (DMEM), fetal bovine serum (FBS), crystal violet, agar, cell proliferation and cytotoxicity assay kit (MTT), and actin antibody were purchased from Sigma-Aldrich (St.Louis, MO, USA). H1299, and H522 cell lines were obtained from the American Type Culture Collection (Manassas, VA, USA). PC-9 cell line was kindly provided by Dr. Shen (Jilin University, China). A cell counting kit-8 (MTT kit) and transwell were obtained from Biosharp (Wuhan, China) and BD Biosciences (Franklin Lakes, NJ, USA), respectively. VECTASTAIN ABC Kits (HRP) from Vector Laboratories (Burlingame, CA, USA). Antibodies against PGK1, ubiquitin, ADRM1, Flag, pro-SFTPB, HRP-conjugated anti-mouse IgG, FITC-conjugated anti-mouse IgG, and Texas red-conjugated anti-mouse IgG were purchased from Proteintech (Rosemont, IL, USA). Antibodies against to p-Akt (Ser 473), Akt, UCH37, hRpn2, p-mTOR, and mTOR were obtained from Abcam (Cambridge, UK). Pro-SFTPB elisa kit was purchased from Shanghai Baiyi Biotechnology Co., Ltd. (Shanghai, China). siRNAs of PGK1, ADRM1 and UCH37 genes were obtained from Shanghai GeneBio Co., Ltd (Shanghai, China) and shRNAs of pro-SFTPB were obtained from Hanbio Biotechnology Co., Ltd. (Shanghai, China). The target sequences of siRNA and shRNA were summarized in Additional file 1: Table S2.

### Cell culture and specimens

All cells were cultured in DMEM supplemented with 10% FBS at 37 ℃ in an atmosphere of 95% air and 5% CO_2_. Human specimens were collected at Daping Hospital of Army Medical University during surgery under a protocol approved by the ethical review committees. Characteristics of the patients were summarized in Additional file 1: Table S3 and S4.

### Cell viability, soft agar, and transwell assays

Analysis was performed 24 h after transfection of cells with indicated constructs or nucleotides. For cell viability assay, cells were seeded into 96-well plates at a density of 5 × 10^3^ cells per well, and cell viability was measured at indicated time using MTT assay kit according to the manufacturer`s protocol. For transwell assay, cells in growth medium without FBS were plated in the upper wells of 24-well chamber at density of 1 × 10^4^ cells per well. The lower wells of chamber contained cell growth medium supplemented with 10% FBS. After 24 h of seeding, cells in the lower side of the chamber were fixed, stained and counted. For soft agar assay, cells were mixed in 0.5 ml 0.35% agar in growth medium and plated on the top of a solid layer of 0.8% agar in growth medium at a density of 3000 cells per well (6-well plate). Colonies were counted 10 days later.

### Western blot, co-immunoprecipitation (Co-IP), immunohistochemistry (IHC) and immunofluorescence (IF) analysis

Western blot, Co-IP, IHC, and IF were performed as described previously [15]. After IF analysis, the colocalization between proteins were measured using Image J 1.53t (National Institutes of Health, USA).

### Proteomics analysis

H1299 cells were transfected with indicated plasmids. After 72 h of transfection, cells were harvested and extracted proteins, and then subjected to analysis. Proteomics analysis was performed as described [16].

### Animal experiments

6-weeks old male nude mice were used in this study. For tumorigenesis experiment, 1 × 10^6^ indicated cells in 1 ml PBS were injected subcutaneously on the back of mice. After, 3 weeks, mice were sacrificed and the tumors were weighed. For lung metastasis experiment, 1.5 × 10^6^ indicated cells in 1 ml PBS were injected into mice via tail vein. After, 3 weeks, mice were sacrificed and the lungs were collected and the tumor nodules on the lung surface were counted under a microscopy.

### Statistical analysis

Data were presented as mean ± standard deviation and the differences between groups were considered statistically significant at a *p* value of less than 0.05. The differences between two groups were analyzed by a student *t* test using SAS statistical software (SAS Institute). The survival rate of patients with early-stage NSCLC was calculated by Kaplan–Meier survival analysis.

## Results

### Low expression of pro-SFTPB in primary tumors compared to adjacent tissues is associated with recurrence in early-stage LUAD

**Fig. 1:**
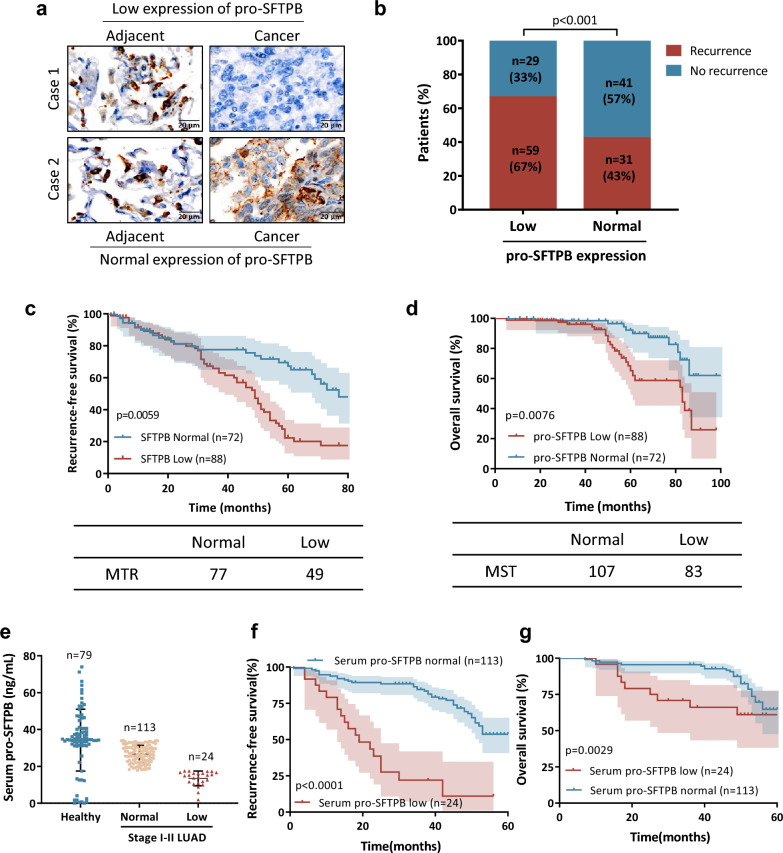
Low expression of pro-SFTPB is associated with progression of early-stage lung adenocarcinoma (LUAD). **a** pro-SFTPB expression in the primary tumors of patients with early-stage LUAD and in the tissues adjacent to these tumors was measured by immunohistochemistry (n = 160). **b**-**d** Low expression of pro-SFTPB compared to the adjacent tissue correlated with high recurrence (**b**), lower recurrence-free survival (**c**), and lower overall survival in patients with early-stage LUAD (n = 160) (**d**). **e** Patients with early-stage LUAD whose serum pro-SFTPB concentrations were within the range of concentrations (measured as mean ± SEM) found in healthy individuals were placed in the normal pro-SFTPB group, and patients whose serum pro-SFTPB concentrations fell below that range were placed in the low pro-SFTPB group. **f**-**g** Kaplan‒Meier survival analysis showed that low levels of serum pro-SFTPB were closely correlated with lower recurrence-free survival (**f**) and lower overall survival of patients with early-stage LUAD (**g**). *P* values were calculated using the log-rank test. MTR, median time of recurrence; MST, median time of survival

Based on their immunohistochemistry (IHC) results, early-stage lung adenocarcinoma (LUAD) patients who showed no difference in pro-SFTPB expression in the tumor and its adjacent tissue were placed in the pro-SFTPB normal expression group, and patients in whom pro-SFTPB expression was lower in the tumor than in the adjacent tissue were placed in the pro-SFTPB low expression group (Fig. 1a). We then analyzed the correlation between pro-SFTPB expression and progression of early-stage LUAD. The results showed that the group with low pro-SFTPB expression had a significantly higher recurrence rate (Fig. 1b), shorter recurrence-free survival (RFS) (Fig. 1c) and shorter overall survival (OS) (Fig. 1d) than the group with normal pro-SFTPB expression. Specifically, the median time of recurrence (MTR) and median survival time (MST) of the pro-SFTPB expression group were 77 months and 107 months, respectively, while the MTR and MST of the low pro-SFTPB expression group were 49 months and 83 months, respectively. Consistent with this, RNA sequencing data showed that early-stage LUAD patients who experienced recurrence within 24 months after surgery had lower levels of expression of SFTPB than patients who experienced no recurrence within 5 years after surgery (Additional file 1: Fig. S1). In addition, we investigated the clinical significance of low levels of serum pro-SFTPB in the progression of early-stage LUAD. Patients with early-stage LUAD whose serum pro-SFTPB concentration was within the range of pro-SFTPB concentrations in healthy people (Mean ± SE) were placed in the normal pro-SFTPB group, and patients whose serum pro-SFTPB concentration was lower than the pro-SFTPB concentration in healthy people were placed in the low pro-SFTPB group (Fig. 1e). Our data show that, among these patients with early-stage LUAD, the low pro-SFTPB group had both lower RFS (*p* < 0.0001) (Fig. 1f) and lower OS (*p* = 0.0029) (Fig. 1g) than the normal pro-SFTPB group. Taken together, our findings suggest that low expression of pro-SFTPB is associated with poor prognosis and that it may be involved in the stimulation of recurrence in early-stage LUAD patients.

### Downregulation of pro-SFTPB expression enhances the metastasis and tumorigenicity of NSCLC cells

**Fig. 2:**
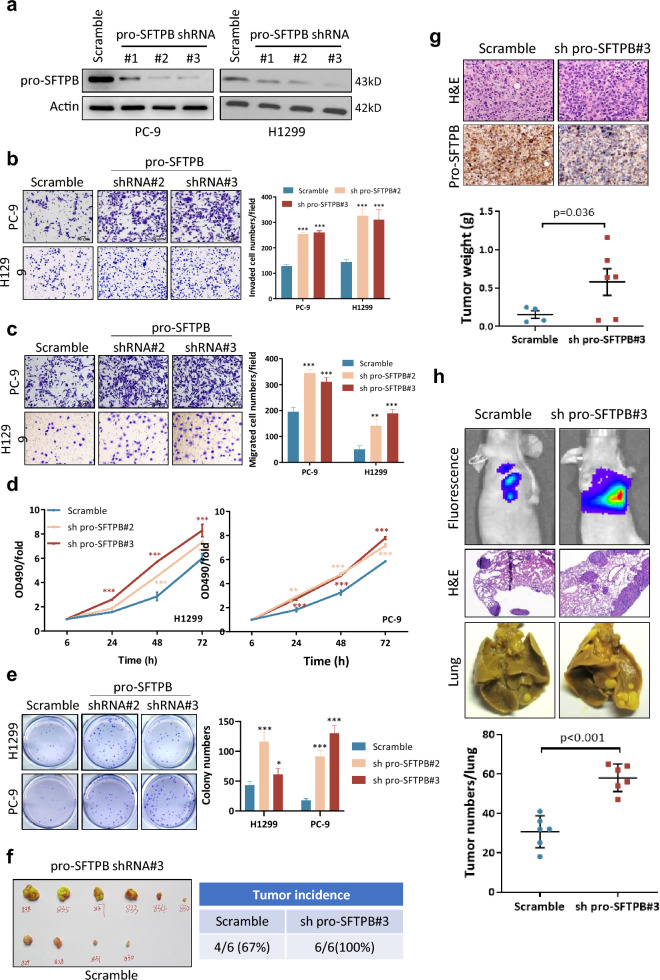
Downregulation of pro-SFTPB expression stimulates NSCLC progression. **a** The indicated NSCLC cells were transfected with pro-SFTPB shRNA, and pro-SFTPB expression levels were measured by Western blotting 72 h after transfection. **b**-**c** Downregulation of pro-SFTPB expression stimulates invasion (**b**) and migration (**c**) by NSCLC cells. Forty-eight hours after transfection with pro-SFTPB shRNA, the cells were subjected to invasion and migration assays. **d** Downregulation of pro-SFTPB expression increased cell viability. Twenty-four hours after transfection with SFTPB shRNA, the cells were reseeded in 96-well plates, and MTT assays were performed. **e** Downregulation of pro-SFTPB expression stimulates colony formation in soft agar by NSCLC cells. The cells were subjected to analysis 24 h after transfection with pro-SFTPB shRNA. **f**-**g** Subcutaneous xenograft model experiment (n = 6/group) showing that downregulation of pro-SFTPB stimulated tumorigenicity (**f**) and tumor growth (**g**) in H1299 cells. **h** Lung metastasis analysis showing that downregulation of pro-SFTPB expression stimulated tumor metastasis (n = 6/group). sh pro-SFTPB, shRNA of pro-SFTPB. * indicates comparison with the scrambled control group. *, p < 0.05; **, p < 0.01; ***, p < 0.001

Next, we investigated whether downregulation of pro-SFTPB directly promotes metastasis and tumorigenicity in NSCLC cells. NSCLC cell lines that highly expressed pro-SFTPB were selected (Additional file 1: Fig. S2), and pro-SFTPB expression in these cells was silenced using shRNAs of pro-SFTPB (Fig. 2a). Our data show that downregulation of pro-SFTPB expression significantly promoted NSCLC cell invasion (Fig. 2b), migration (Fig. 2c), viability (Fig. 2d), and colony formation in soft agar (Fig. 2e). These in vitro results were confirmed in animal models. Animal experiments showed that whereas subcutaneous injection of 1 × 10^6^ H1299 cells resulted in tumor formation in 67% of mice within 3 weeks, injection of H1299 cells in which pro-SFTPB had been downregulated resulted in tumor formation in 100% of the animals (Fig. 2f). In addition, downregulation of pro-SFTPB dramatically stimulated NSCLC growth in subcutaneous xenograft models (Fig. 2f, g). Furthermore, an experiment in which a lung metastasis model was used showed that downregulation of pro-SFTPB significantly increased the number of tumor colonies in the lungs (Fig. 2h). In contrast, ectopic expression of pro-SFTPB significantly inhibited growth and metastasis of NSCLC with low expression of pro-SFTPB (Additional file 1: Fig. S3). Together, these findings suggest that pro-SFTPB negatively contribute to NSCLC cell metastasis and tumorigenicity.

### Downregulation of pro-SFTPB promotes NSCLC progression by activating the Akt pathway

**Fig. 3:**
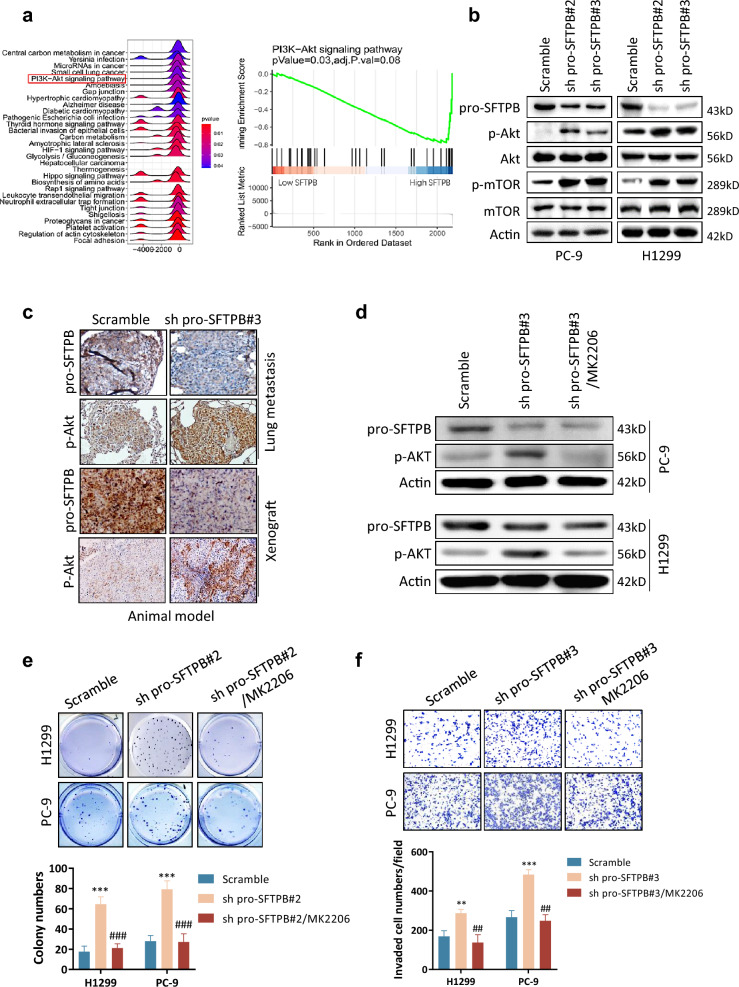
Downregulation of pro-SFTPB expression stimulates NSCLC progression by activating the Akt pathway. **a** Gene Set Enrichment Analysis using the TCGA dataset showing that the SFTPB level is negatively correlated with the Akt pathway in early-stage LUAD. **b** Western blot analysis showing that downregulation of pro-SFTPB activated the Akt/mTOR signaling pathway in NSCLC cells. The cells were subjected to analysis 72 h after transfection with pro-SFTPB shRNA. **c** Immunohistochemistry analysis showing that downregulation of pro-SFTPB increased Akt phosphorylation (Ser 473) in NSCLC tissues in subcutaneous xenograft models and lung metastasis models. **d** The Akt inhibitor MK2206 significantly inhibited the silencing of the pro-SFTPB-induced upregulation of Akt phosphorylation in NSCLC cells. The indicated NSCLC cells were transfected with shRNA pro-SFTPB shRNA. Forty-eight hours after transfection, the cells were treated with 5 µM MK2206 for 12 h and then subjected to Western blotting. **e–f** Soft agar (**e**) and invasion (**f**) assays showing that inhibition of the Akt pathway by the Akt inhibitor MK2206 significantly suppresses the downregulation of pro-SFTPB-stimulated soft agar colony formation and invasion by NSCLC cells. Forty-eight hours after transfection with pro-SFTPB shRNA (sh pro-SFTPB), the cells were treated with 5 µM MK2206 for 12 h and then subjected to analysis. *, compared to the scrambled control group; #, compared to the pro-SFTPB downregulation group. *, p < 0.05; **, p < 0.01; ***, p < 0.001; ^#^, p < 0.05; ^##^, p < 0.01; ^###^, p < 0.001

To investigate the mechanism by which downregulation of pro-SFTPB promotes NSCLC progression, we performed gene set enrichment analysis (GSEA) using an early-stage NSCLC dataset from the TCGA database. The GSEA results showed that SFTPB expression correlates negatively with activity of the Akt signaling pathway in early-stage NSCLC (Fig. 3a). Consistent with this, in vitro experiments showed that downregulation of pro-SFTPB expression significantly increased the phosphorylation of both Akt and its downstream protein mTOR in NSCLC cells (Fig. 3b). The promoting effect of pro-SFTPB downregulation on Akt phosphorylation was further confirmed by IHC in tumors from xenografted mice and lung metastasis animal models (Fig. 3c). Notably, inhibition of Akt activity by the Akt inhibitor MK2206 (Fig. 3d) significantly suppressed the increase in colony formation in soft agar (Fig. 3e) and the increased invasion by NSCLC cells (Fig. 3f) that were otherwise induced by downregulation of pro-SFTPB expression. These findings indicate that downregulation of pro-SFTPB expression promotes NSCLC progression by activating the Akt pathway.

### Downregulation of pro-SFTPB expression activates the Akt pathway by upregulating PGK1 in NSCLC

**Fig. 4:**
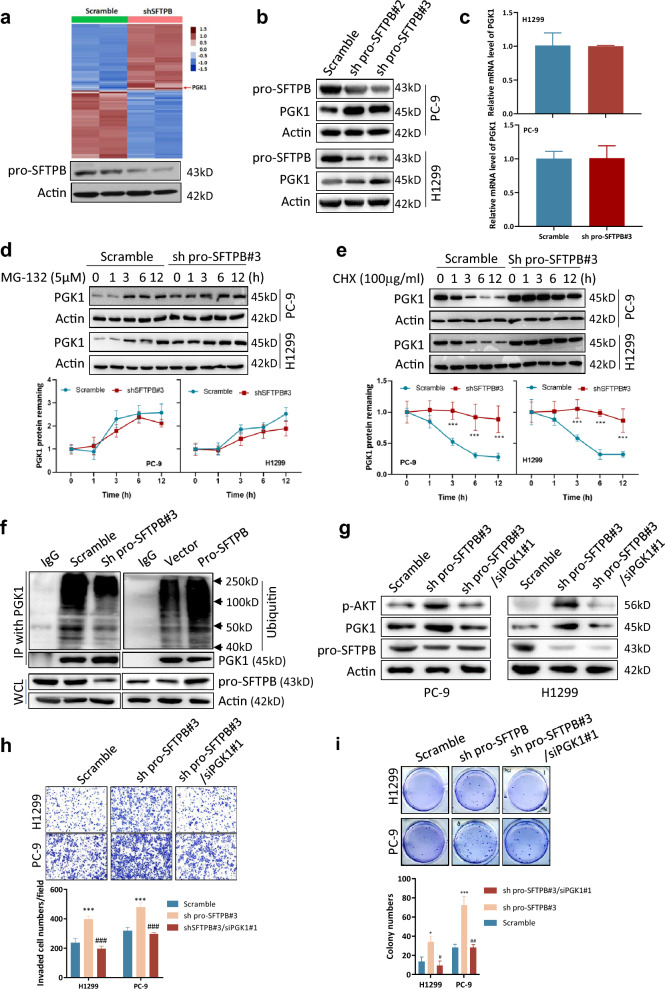
Downregulation of pro-SFTPB expression activates the Akt pathway through upregulation of PGK1 in NSCLC cells. **a** Proteomics analysis showing that downregulation of pro-SFTPB upregulates PGK1 expression. H1299 cells were subjected to analysis 72 h after transfection with scrambled RNA or pro-SFTPB shRNA. **b** Western blot analysis showing that downregulation of pro-SFTPB upregulates PGK1 protein levels in NSCLC cells. The cells were subjected to Western blot analysis 72 h after transfection. **c** qRT‒PCR analysis showing that downregulation of pro-SFTPB did not affect PGK1 mRNA levels in NSCLC cells. The cells were subjected to qRT‒PCR analysis 72 h after transfection with scrambled RNA or pro-SFTPB shRNA. **d** Protein half-life analysis using cycloheximide (CHX) showing that downregulation of pro-SFTPB prolonged the half-life of PGK1 protein. Forty-eight hours after transfection with scrambled RNA or pro-SFTPB shRNA, the cells were treated with 100 µg/ml CHX for the indicated times, and PGK1 protein levels were measured by Western blotting. **e** Downregulation of pro-SFTPB does not affect PGK1 protein synthesis. Forty-eight hours after transfection with scrambled RNA or SFTPB shRNA, the cells were treated with 5 µM MG-132 for the indicated times, and PGK1 protein levels were measured by Western blotting. **f** Ubiquitination analysis showing that pro-SFTPB positively regulates PGK1 ubiquitination. Seventy-two hours after transfection with the indicated constructs, PGK1 proteins were isolated by IP, and ubiquitinated PGK1 was measured by Western blotting. WCL, whole cell lysate. **g** Western blot analysis showing that silencing of PGK1 inhibits the pro-SFTPB-induced upregulation of Akt phosphorylation. Seventy-two hours after transfection with the indicated constructs, the cells were subjected to Western blot analysis. **h** Silencing of PGK1 inhibited the downregulation of pro-SFTPB-stimulated invasion by NSCLC cells. Seventy-two hours after transfection with the indicated constructs, the cells were subjected to invasion analysis. **i** Silencing of PGK1 inhibited the downregulation of pro-SFTPB-stimulated colony formation in soft agar by NSCLC cells. Seventy-two hours after transfection with the indicated constructs, the cells were subjected to soft agar colony formation assays. Abbreviations: sh pro-SFTPB, shRNA of pro-SFTPB; siPGK1, siRNA of PGK1. *, compared to the scrambled control group; #, compared to the pro-SFTPB downregulation group. *, p < 0.05; **, p < 0.01; ***, p < 0.001; ^#^, p < 0.05; ^##^, p < 0.01; ^###^, p < 0.001

To investigate how downregulation of pro-SFTPB activates the Akt pathway, we performed a proteomics analysis of control cells and NSCLC cells in which pro-SFTPB had been downregulated. As shown in Fig. 4a and Table S1, many proteins were upregulated after downregulation of pro-SFTPB expression in NSCLC cells. We chose PGK1 for further analysis because it is an activator of the Akt pathway [17] and stimulates tumor progression by promoting glycolysis [18]. The effect of downregulation of pro-SFTPB expression on PGK1 expression was further examined at the mRNA and protein levels. Our results show that downregulation of pro-SFTPB upregulated the protein level of PGK1 (Fig. 4b) but that it did not affect the mRNA level of PGK1 in NSCLC cells (Fig. 4c). In addition, downregulation of pro-SFTPB expression increased the half-life of PGK1 protein when the cells were treated with the protein synthesis inhibitor cycloheximide (CHX) (Fig. 4d) but did not affect PGK1 level when the cells were treated with the proteasome inhibitor MG-132 (Fig. 4e). Downregulation of pro-SFTPB expression also reduced PGK1 ubiquitination, while overexpression of pro-SFTPB increased it (Fig. 4f). These findings suggest that downregulation of pro-SFTPB expression upregulates PGK1 expression at the posttranscriptional level by inhibiting its ubiquitination. Notably, downregulation of PGK1 expression by siRNAs (Additional file 1: Fig. S4) inhibited the stimulation of Akt phosphorylation (Fig. 4g), invasion (Fig. 4h), and soft agar colony formation (Fig. 4i) by NSCLC cells that otherwise occurred after downregulation of pro-SFTPB expression, suggesting that downregulation of pro-SFTPB promotes Akt pathway-regulated NSCLC progression by upregulating PGK1 levels.

### Downregulation of pro-SFTPB upregulates PGK1 protein levels through ADRM1/UCH37-mediated deubiquitination of PGK1

**Fig. 5:**
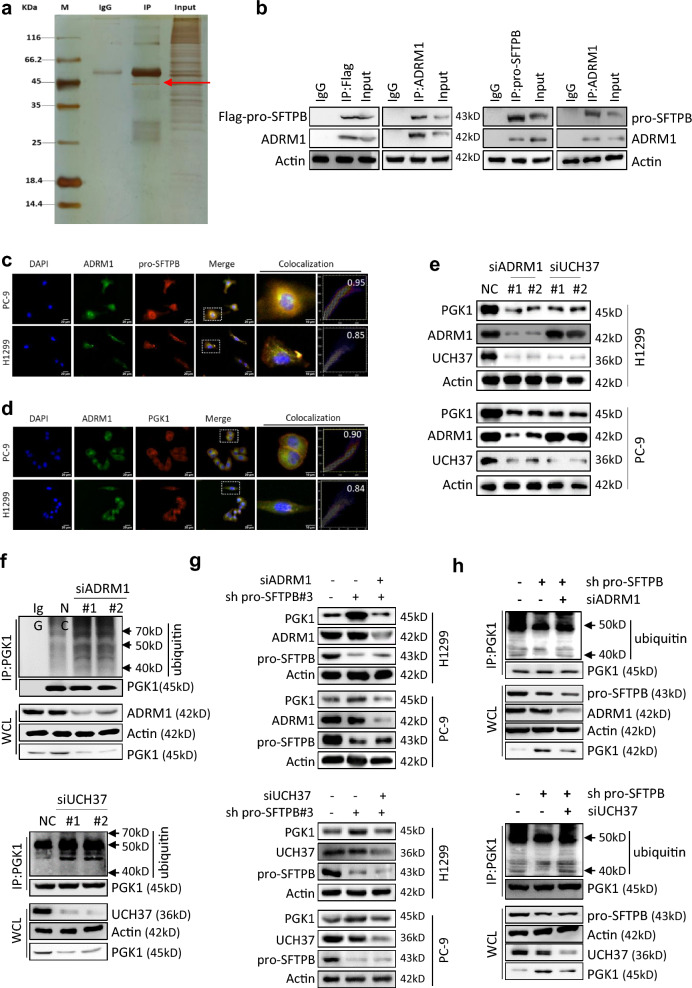
Downregulation of pro-SFTPB expression upregulates PGK1 expression through ADRM1. **a** Silver staining showing the proteins that interact with pro-SFTPB in H1299 cells. Proteins that were bound to pro-SFTPB were pulled down by Co-IP using a pro-SFTPB antibody, separated by electrophoresis, and silver stained. The red arrow indicates the ADRM1 protein. **b** Co-IP analysis showing that pro-SFTPB interacts with ADRM1 in NSCLC cells. Flag-tagged pro-SFTPB was overexpressed in H1650 cells. Seventy-two hours after transfection, cell lysates were prepared, and co-IP was performed using antibodies against Flag or ADRM1. To detect the interaction between endogenous pro-SFTPB and ADRM1 proteins, the protein was extracted from H1299 cells and co-IP was performed using antibodies against pro-SFTPB or ADRM1. **c** Colocalization of pro-SFTPB and ADRM1 in NSCLC cells was demonstrated by immunofluorescence (IF) analysis. **d** Colocalization of PGK1 and ADRM1 in NSCLC cells was demonstrated by IF analysis. **e** Silencing of ADRM1 or UCH37 decreased PGK1 protein levels in NSCLC cells. The cells were subjected to Western blot analysis 72 h after transfection with the indicated siRNAs. **f** Downregulation of ADRM1 or UCH37 increases PGK1 ubiquitination in NSCLC cells. The cells were subjected to analysis 72 h after transfection with the indicated siRNAs. Co-IP was performed using an antibody against PGK1, and ubiquitinated PGK1 was detected by Western blotting. **g** Silencing of ADRM1 or UCH37 suppresses the pro-SFTPB-induced upregulation of PGK1 protein levels. Cells were subjected to Western blot analysis 72 h after transfection with the indicated siRNAs. **h** Silencing of ADRM1 or UCH37 suppresses the downregulation of pro-SFTPB-induced inhibition of PGK1 ubiquitination. The cells were subjected to analysis 72 h after transfection with the indicated siRNAs. Co-IP was performed using an antibody against PGK1, and ubiquitinated PGK1 was detected by Western blotting. sh pro-SFTPB, shRNA of pro-SFTPB; siADRM1, siRNA of ADRM1; siUCH37, siRNA of UCH37

To investigate how pro-SFTPB regulates PGK1 ubiquitination, we collected proteins bound to pro-SFTPB by coimmunoprecipitation (Co-IP) and identified them by mass spectrometry (Fig. 5a). The results of the mass spectrometry analysis showed that ADRM1 may be a binding partner of pro-SFTPB in NSCLC cells (Fig. 5a). The interaction (Fig. 5b) and colocalization (Fig. 5c) of ADRM1 and pro-SFTPB in NSCLC cells were further confirmed by co-IP and immunofluorescence (IF), respectively. ADRM1 is an ubiquitin receptor that is involved in protein deubiquitination by activating the deubiquitinating enzyme UCH37 (ubiquitin carboxy-terminal hydrolase 37) [19]. Importantly, we found colocalization of ADRM1 and PGK1 (Fig. 5d), and silencing of ADRM1 or UCH37 decreased PGK1 protein levels (Fig. 5e) and increased PGK1 ubiquitination in NSCLC cells (Fig. 5f), suggesting that the ADRM1/UCH37 axis is involved in the regulation of PGK1 deubiquitination. Silencing of ADRM1 or UCH37 also suppressed the downregulation of pro-SFTPB-induced upregulation of PGK1 protein (Fig. 5g) and downregulation of PGK1 ubiquitination (Fig. 5h). Together, these findings suggest that the ADRM1/UCH37 axis is involved in the regulatory effect of pro-SFTPB on the ubiquitination of PGK1 in NSCLC.

### Pro-SFTPB inhibits ADRM1/UCH37 complex formation by inhibiting hRpn2 binding to ADRM1

**Fig. 6:**
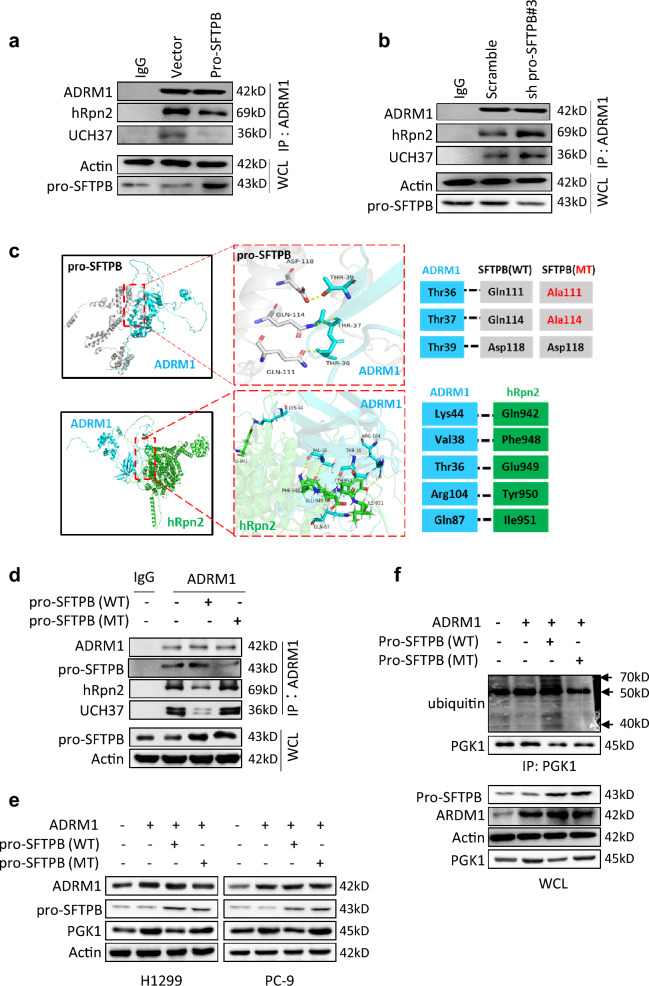
pro-SFTPB inhibits ADRM1-mediated PGK1 deubiquitination by binding to ADRM1. **a**-**b** Overexpression of pro-SFTPB reduces the interaction among ADRM1, hRpn2 and UCH37 in H1299 cells (**a**), while downregulation of pro-SFTPB expression increases it (**b**). Seventy-two hours after transfection, an anti-ADRM1 antibody was used to perform co-IP, and the indicated proteins were detected by Western blotting. **c** Predicted interaction residues between pro-SFTPB and ADRM1. **d** Co-IP analysis showing that overexpression of a mutant form of pro-SFTPB that binds to ADRM1 does not affect binding of ADRM1, hRpn2 and UCH37 in H1299 cells. Seventy-two hours after transfection, an anti-ADRM1 antibody was used to perform co-IP, and the indicated proteins were measured by Western blotting. **e**–**f** Overexpression of wild-type pro-SFTPB suppresses ADRM1-induced upregulation of PGK1 **(e)** and PGK1 deubiquitination **(f)**, whereas mutant pro-SFTPB does not. Seventy-two hours after transfection, H1299 cells were subjected to Co-IP and Western blot analysis. WCL, whole cell lysate

To investigate the mechanism by which pro-SFTPB regulates PGK1 deubiquitination through the ADRM1/UCH37 axis, we examined whether pro-SFTPB affects the formation of complexes between ADRM1 and its partner proteins. Previous studies have shown that hRpn2 activates ADRM1 by binding directly to ADRM1 and that activated ADRM1 interacts with the deubiquitinase UCH37 and then exerts its deubiquitination effect [20]. As shown in Fig. 6a and b, pro-SFTPB negatively regulated the binding of hRpn2 and UCH37 to ADRM1. In addition, mimetic molecular docking between pro-SFTPB and ADRM1 by HDOCK showed that pro-SFTPB may bind to the N-terminal Pru (pleckstrin-like receptor for ubiquitin) domain (Fig. 6c) of ADRM1; this domain is important in the activation of ADRM1 by hRpn2 through physical binding (Fig. 6c) [20]. Based on these results, we hypothesized that binding of pro-SFTPB to ADRM1 suppresses hRpn2 binding to ADRM1 and thereby inhibits ADRM1 activation and the interaction between ADRM1 and UCH37. As expected, unlike overexpression of wild-type pro-SFTPB, overexpression of pro-SFTPB mutated at the ADRM1 binding site did not affect the interaction of ADRM1 with hRpn2 or the interaction of ADRM1 with UCH37 (Fig. 6d). Additionally, overexpression of mutated pro-SFTPB did not inhibit ADRM1-induced upregulation of PGK1 (Fig. 6e) and downregulation of PGK1 ubiquitination (Fig. 6f). Taken together, these findings suggest that pro-SFTPB inactivates ADRM1 by blocking the binding of hRpn2 and UCH37 to ADRM1, thereby inhibiting the deubiquitination of PGK1 by ADRM1 and ultimately leading to ubiquitination and degradation of PGK1.

### Clinical correlation between pro-SFTPB expression and PGK1 and Akt activation in early-stage NSCLC

**Fig. 7:**
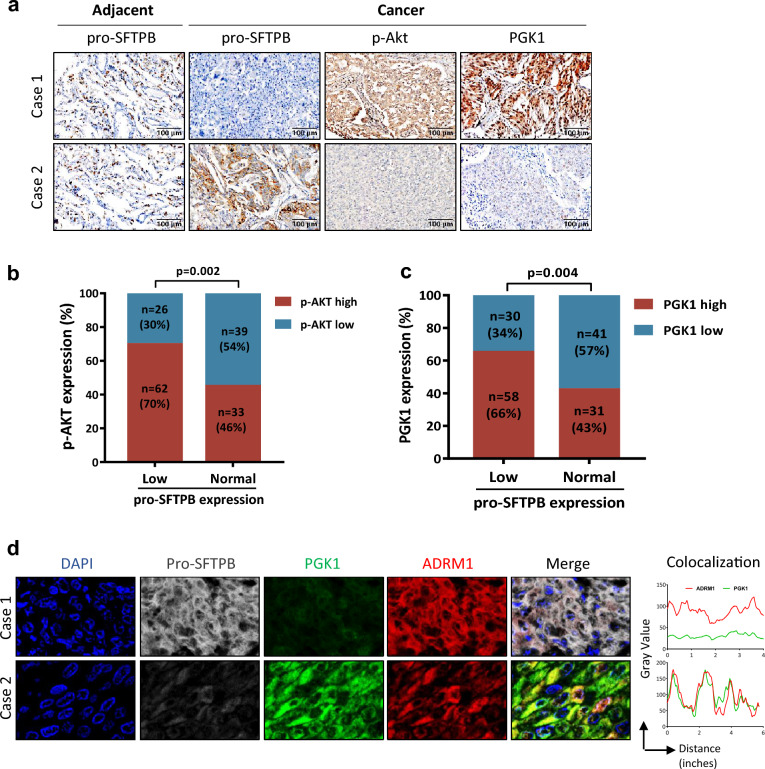
Correlation between the expression of pro-SFTPB and its target genes in clinical samples of early-stage lung adenocarcinoma tissues (LUAD). **a** Immunohistochemistry analysis of pro-SFTPB, p-Akt and PGK1 expression in early-stage LUAD. **b** The correlation between the expression of pro-SFTPB and that of p-Akt was determined in 160 primary tumors from patients with early-stage LUAD. **c** The correlation between the expression of pro-SFTPB and that of PGK1 was determined in 160 primary tumors from patients with early-stage LUAD. **d** The effect of pro-SFTPB level on the expression and colocalization of PGK1 and ADRM1 were measured in clinical samples from patients with early-stage LUAD

Finally, we used clinical samples to examine whether low expression of pro-SFTPB in tumors correlates with the expression of PGK1 and p-AKT in patients with early-stage LUAD. The expression of pro-SFTPB, PGK1 and p-Akt in clinical samples was examined by IHC (Fig. 7a). As shown in Fig. 7a, c, a correlation study of 160 early-stage LUAD clinical samples showed that low expression of pro-SFTPB correlated significantly with high expression of PGK1 and p-Akt in early-stage LUAD. Specifically, 70% (62 cases) and 66% (58 cases) of the samples that showed low pro-SFTPB expression showed high expression of p-Akt (Fig. 7b) and PGK1, respectively (Fig. 7c). In addition, consistent with the results of in vitro experiments, pro-SFTPB not only decreased the expression of PGK1 but also blocked the binding of PGK1 to ADRM1 in early-stage LUAD tissues (Fig. 7d).

## Discussion

Treatment failure in patients with early-stage NSCLC is mainly due to recurrence. Therefore, it is very important to understand the molecular mechanisms that drive or promote recurrence of lung cancer and to identify predictors of early-stage NSCLC recurrence. In the present study, we evaluated the possibility that low expression of pro-SFTPB in cancer tissue compared to adjacent tissue is a predictor of early-stage NSCLC recurrence and elucidated the mechanism by which downregulation of pro-SFTPB expression promotes early-stage NSCLC recurrence.

The effects of pro-SFTPB on lung cancer development and metastasis have been studied previously. However, an association between pro-SFTPB expression and recurrence of early-stage NSCLC has not been reported. In this study, we clearly demonstrated through a series of in vitro and in vivo experiments that downregulation of pro-SFTPB expression significantly promotes the metastatic ability and tumorigenicity of NSCLC cells. Previous studies support our results. Taguchi et al. reported that undetectable plasma pro-SFTPB was associated with higher lung cancer risk [14]. Lee et al. reported that downregulation of SFTPB expression increased the migration of NSCLC cells, while ectopic expression of SFTPB inhibited tumor growth and EMT in an NSCLC xenograft model [21]. Notably, in the present work we used our clinical sample cohort to clearly demonstrate that low expression of pro-SFTPB in NSCLC tissue compared to adjacent tissues correlates significantly with recurrence and poor prognosis in early-stage NSCLC patients, suggesting that low expression of pro-SFTPB in cancer tissue compared to adjacent tissue has potential as a predictor of recurrence and poor prognosis in early-stage NSCLC. However, study of a large cohort is required to validate our findings before clinical application.

We then elucidated the mechanism by which downregulation of pro-SFTPB promotes recurrence of early-stage NSCLC. Accumulating evidence shows that the Akt pathway serves as a central regulator in tumorigenesis, metastasis and drug resistance through promoting cancer stemness, proliferation, survival, migration and metabolism of cancer cells [22–25]. Thus, Akt pathway also is important therapeutic target for cancer treatment [3, 23, 26]. In fact, several inhibitors of the Akt signaling pathway are currently in clinical trials [3, 5]. Importantly, activation or inhibition of the Akt pathway is one of the important mechanisms by which oncogenes and tumor suppressor genes exert their role [24, 25, 27]. For example, Hsu et al. reported that huntingtin-interacting protein 1 is an early-stage prognostic biomarker of lung adenocarcinoma and that it suppresses metastasis by inhibiting the Akt pathway [27]. Li et al. reported that OTUB2 expression is closely correlated with NSCLC recurrence and that it plays an oncogenic role by activating the Akt pathway [28]. Here, we used clinical sample analysis and a series of experiments to clearly indicate that low expression of pro-SFTPB causes Akt pathway activation and cancer recurrence in patients with early-stage NSCLC. Importantly, inhibition of the Akt pathway by an Akt inhibitor blocked the stimulation of NSCLC cell metastasis and tumorigenicity that was otherwise induced by downregulation of pro-SFTPB expression. Together, these findings suggest that downregulation of pro-SFTPB stimulates recurrence of early-stage NSCLC by activating the Akt pathway.

Further, we elucidated the mechanism by which pro-SFTPB regulates the Akt pathway. Previous studies have shown that many genes can activate Akt signaling through various molecular mechanisms [24, 25], including PGK1^22^. According to He et al. report PGK1 activate Akt through inducing CXCR4-mediated phosphorylation of Akt, indicating that PGK1 is a activator of Akt signaling pathway [17, 29]. In addition, studies show the level of PGK1 protein is controlled by ubiquitination [17]. In this study, we found that pro-SFTPB reduces PGK1 protein levels by increasing PGK1 ubiquitination, thereby inhibiting the Akt pathway. Here, we demonstrate for the first time that ADRM1 is involved in the regulation of PGK1 deubiquitination and that pro-SFTPB regulates PGK1 protein levels by interacting with ADRM1. ADRM1 is a proteasome ubiquitin receptor that stimulates protein deubiquitination by activating the deubiquitinating enzyme UCH37 by directly binding to UCH37 [30] and positively regulating UCH37 expression [31, 32]. According to Chen et al., the ubiquitin- and UCH37-binding domains of ADRM1 interact with each other, rendering the ADRM1 inactive [20]. However, the proteasome scaffolding protein hRpn2 abrogates these interdomain interactions of ADRM1 by physically binding to the ADRM1 N-terminal Pru (pleckstrin-like receptor for ubiquitin) domain, thus activating ADRM1 for ubiquitin binding and UCH37 binding [20]. We also demonstrate that pro-SFTPB inhibits the interaction of ADRM1 with hRpn2 by binding directly to ADRM1 and that the interaction between pro-SFTPB and ADRM1 also inhibits the interaction between ADRM1 and UCH37. Taken together, these findings indicate that pro-SFTPB inactivates ADRM1 by inhibiting the interaction between ADRM1 and hRpn2 by binding to ADRM1; this inhibits deubiquitination of PGK1 by ADRM1 and accelerates degradation of PGK1, ultimately inhibiting PGK1-mediated Akt pathway activation.

**Fig. 8:**
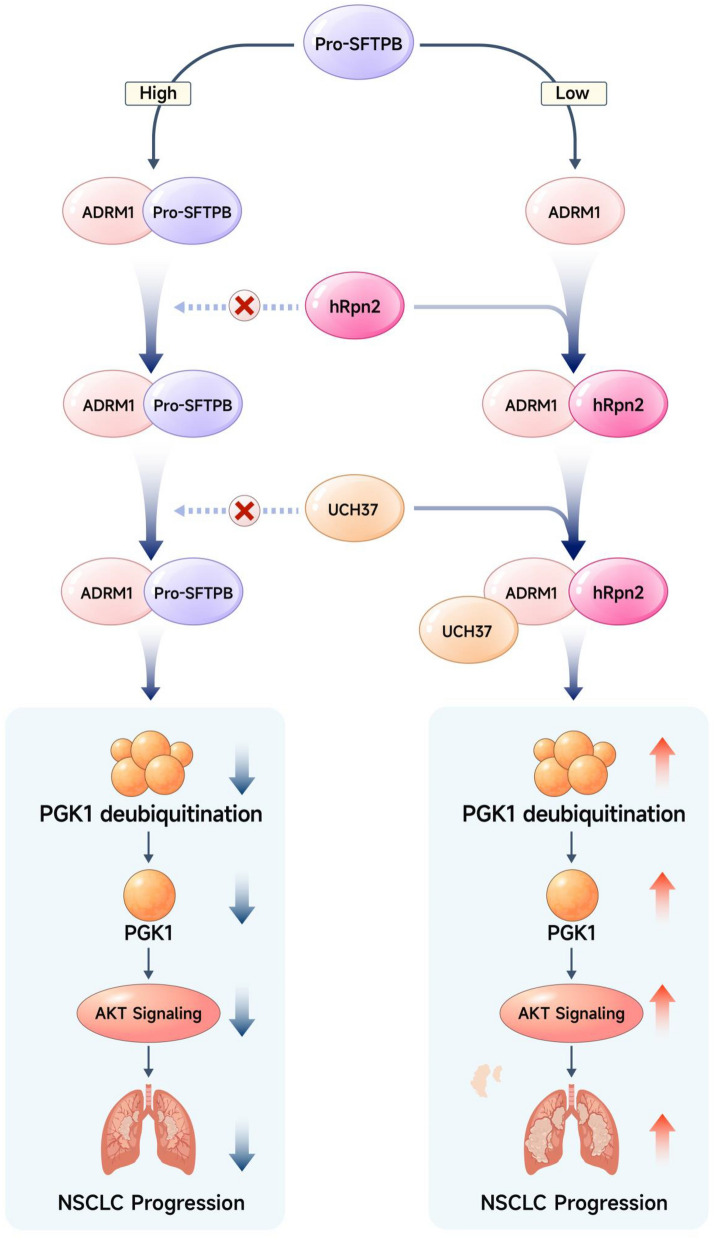
Working model of the action of pro-SFTPB in NSCLC

In conclusion, pro-SFTPB inhibits ADRM1 activation by blocking the binding of hRpn2 to ADRM1 through direct binding to ADRM1, thereby reducing the deubiquitination of PGK1 by ADRM1, which accelerates the ubiquitination and degradation of PGK1 and ultimately inhibits the activation of the PGK1-regulated Akt pathway in NSCLC. Thus, downregulation of pro-SFTPB leads to hyperactivation of the PGK1-mediated Akt pathway, a change that promotes the recurrence of early-stage NSCLC (Fig. 8). Our findings also indicate that low expression of pro-SFTPB in tumors is a predictor of recurrence and is associated with poor prognosis in patients with early-stage NSCLC.

### Supplementary Information

**Additional file 1: Fig. S1 **RNA sequencing analysis. **a **Heatmap showing differentially expressed genes between primary tumors of stage I lung adenocarcinoma patients with postoperative metastatic recurrence and those with no recurrence. RNA sequencing was performed using primary tumors from stage I lung adenocarcinoma patients who experienced metastatic recurrence within 24 months after the operation (n=7) and from patients who experienced no recurrence within 60 months after the operation (n=7). **b **Genes whose expression was significantly increased in primary tumors from stage I lung adenocarcinoma patients who had not relapsed within 24 months relative to those who had relapsed within 60 months. **Fig. S2 **Measurement of pro-SFTPB expression in normal lung epithelial cells and NSCLC cell lines by Western blotting. HPA-EpiC, human pulmonary alveolar epithelial cell. **Fig. S3 **Overexpression of pro-SFTPB inhibited NSCLC progression in animal models. **a **Vector or pro-SFTPB expression plasmid was transfected into H460 cells. The expression level of pro-SFTPB was measured by Western blot in normal lung epithelial cells and H460 cells that transfected with vector or pro-SFTPB expression plasmid (after 72 hs of transfection). **b **Subcutaneous xenograft model experiment (n=5/group) showing that overexpression of pro-SFTPB inhibited tumor growth. Subcutaneously inject 1 × 107 H460 cells in 1ml PBS into the back of each nude mouse. Tumors were collected and weighted after 1 month of cell injection. (**c**) Lung metastasis analysis showing that overexpression of pro-SFTPB expression inhibited tumor metastasis (n=5/group). 1 × 107 H460 cells in 1ml PBS injected into each nude mouse through the tail vein. The lungs were collected after one month of cell injection, and counted the number of tumor on the surface of the lungs. **Fig. S4 **Measurement of PGK1 expression by Western blotting. H1299 and PC-9 cells were transfected with siRNAs against PGK1. The cells were subjected to Western blotting 72 h after transfection. **Table S1**. Proteins affected by pro-SFTPB silencing. **Table S2. **shRNA or siRNA sequences used throughout this study. **Table S3 **Characteristics of patients with early-stage lung adenocarcinoma (For IHC). **Table S4 **Characteristics of patients with early-stage lung adenocarcinoma (for serum).

## Data Availability

The data used to support the findings of this study are available from the corresponding author upon request.
