# Supplementary material for: Downregulation of pro-surfactant protein B contributes to the recurrence of early-stage non-small cell lung cancer by activating PGK1-mediated Akt signaling
Source: Exp Hematol Oncol. 2023 Nov 9;12:94. doi: 10.1186/s40164-023-00455-6 (PMC10633994; doi:10.1186/s40164-023-00455-6)
Supplement: Supplementary file 1 — Additional file 1: Fig. S1 RNA sequencing analysis. a Heatmap showing differentially expressed genes between primary tumors of stage I lung adenocarcinoma patients with postoperative metastatic recurrence and those with no recurrence. RNA sequencing was performed using primary tumors from stage I lung adenocarcinoma patients who experienced metastatic recurrence within 24 months after the operation (n=7) and from patients who experienced no recurrence within 60 months after the operation (n=7). b Genes whose expression was significantly increased in primary tumors from stage I lung adenocarcinoma patients who had not relapsed within 24 months relative to those who had relapsed within 60 months. Fig. S2 Measurement of pro-SFTPB expression in normal lung epithelial cells and NSCLC cell lines by Western blotting. HPA-EpiC, human pulmonary alveolar epithelial cell. Fig. S3 Overexpression of pro-SFTPB inhibited NSCLC progression in animal models. a Vector or pro-SFTPB expression plasmid was transfected into H460 cells. The expression level of pro-SFTPB was measured by Western blot in normal lung epithelial cells and H460 cells that transfected with vector or pro-SFTPB expression plasmid (after 72 hs of transfection). b Subcutaneous xenograft model experiment (n=5/group) showing that overexpression of pro-SFTPB inhibited tumor growth. Subcutaneously inject 1 × 10^7^ H460 cells in 1ml PBS into the back of each nude mouse. Tumors were collected and weighted after 1 month of cell injection. (c) Lung metastasis analysis showing that overexpression of pro-SFTPB expression inhibited tumor metastasis (n=5/group). 1 × 10^7^ H460 cells in 1ml PBS injected into each nude mouse through the tail vein. The lungs were collected after one month of cell injection, and counted the number of tumor on the surface of the lungs. Fig. S4 Measurement of PGK1 expression by Western blotting. H1299 and PC-9 cells were transfected with siRNAs against PGK1. The cells were subjected to Western blotting 7 [file 40164_2023_455_MOESM1_ESM.pdf]

Supplemental information

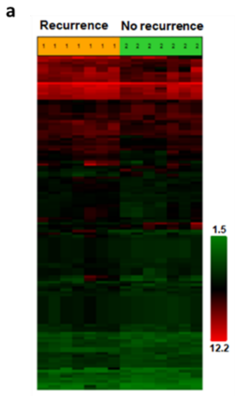

b

Gene	Fold	*p*
SCGB3A2	3.74	0.01
SNORD14D	3.55	0.01
SFTPB	3.24	0.01
RNU6-964P	2.06	0.01
INHBB	2.04	0.03

Fold: No recurrence/Recurrence

Fig. S1 RNA sequencing analysis. **a** Heatmap showing differentially expressed genes between primary tumors of stage I lung adenocarcinoma patients with postoperative metastatic recurrence and those with no recurrence. RNA sequencing was performed using primary tumors from stage I lung adenocarcinoma patients who experienced metastatic recurrence within 24 months after the operation (n=7) and from patients who experienced no recurrence within 60 months after the operation (n=7). **b** Genes whose expression was significantly increased in primary tumors from stage I lung adenocarcinoma patients who had not relapsed within 24 months relative to those who had relapsed within 60 months.

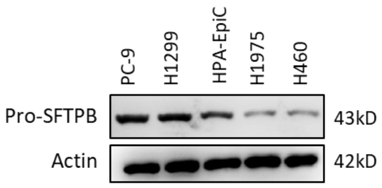

Fig. S2 Measurement of pro-SFTPB expression in normal lung epithelial cells and NSCLC cell lines by Western blotting. HPA-EpiC, human pulmonary alveolar epithelial cell.

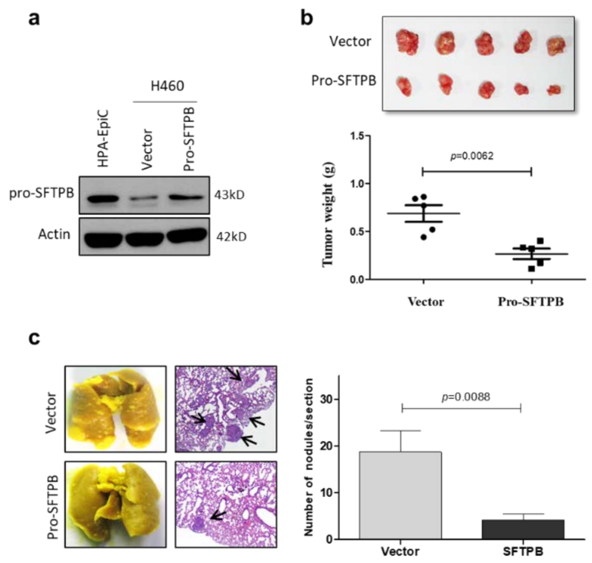

Fig. S3 Overexpression of pro-SFTPB inhibited NSCLC progression in animal models. **a** Vector or pro-SFTPB expression plasmid was transfected into H460 cells. The expression level of pro-SFTPB was measured by Western blot in normal lung epithelial cells and H460 cells that transfected with vector or pro-SFTPB expression plasmid (after 72 hs of transfection). **b** Subcutaneous xenograft model experiment (n=5/group) showing that overexpression of pro-SFTPB inhibited tumor growth. Subcutaneously inject 1×10^7 H460 cells in 1ml PBS into the back of each nude mouse. Tumors were collected and weighted after 1 month of cell injection. (**c**) Lung metastasis analysis showing that overexpression of pro-SFTPB expression inhibited tumor metastasis (n=5/group). 1×10^7 H460 cells in 1ml PBS injected into each nude mouse through the tail vein. The lungs were collected after one month of cell injection, and counted the number of tumor on the surface of the lungs.

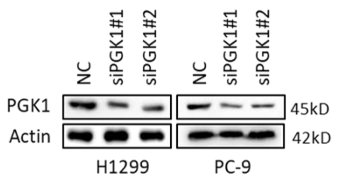

Fig. S4 Measurement of PGK1 expression by Western blotting. H1299 and PC-9 cells were transfected with siRNAs against PGK1. The cells were subjected to Western blotting 72 h after transfection.

Table S1. Proteins affected by pro-SFTPB silencing

Gene Name	Description	Average Scramble	Average sh pro-SFTPB	Scramble/sh pro-SFTPB	P value
DKFZp761K058	Putative uncharacterized protein DKFZp761K058 OS=Homo sapiens GN=DKFZp761K058 PE=2 SV=2	29.2	148.6	5.0890411	3.48E-09
RRBP1	RRBP1 protein OS=Homo sapiens GN=RRBP1 PE=2 SV=1	31.65	142.7	4.5086888	4.44E-08
CRIP1	Cysteine-rich protein 1 OS=Homo sapiens GN=CRIP1 PE=1 SV=3	33	134.05	4.0621212	3.43E-07
ALDH1A1	Retinal dehydrogenase 1 OS=Homo sapiens GN=ALDH1A1 PE=1 SV=2	34.5	134.9	3.9101449	7.01E-07
BASP1	Brain acid soluble protein 1 OS=Homo sapiens GN=BASP1 PE=1 SV=2	39.75	147.15	3.7018868	1.89E-06
SLC39A8	Zinc transporter ZIP8 OS=Homo sapiens GN=SLC39A8 PE=2 SV=1	44.4	164.15	3.6970721	1.94E-06
KIAA1609	KIAA1609 protein, isoform CRA_a OS=Homo sapiens GN=KIAA1609 PE=4 SV=1	38.75	137.7	3.5535484	3.88E-06
AKR1B10	Aldo-keto reductase family 1 member B10 OS=Homo sapiens GN=AKR1B10 PE=1 SV=2	36.95	129.9	3.5155616	4.67E-06
PGK1	Phosphoglycerate kinase 1 OS=Homo sapiens GN=PGK1 PE=1 SV=3	39.35	136.5	3.4688691	5.86E-06
KRT7	Keratin, type II cytoskeletal 7 OS=Homo sapiens GN=KRT7 PE=1 SV=5	39.35	135.15	3.4345616	6.94E-06
CPLX2	Complexin-2 OS=Homo sapiens GN=CPLX2 PE=2 SV=2	39.35	132.1	3.3570521	1.02E-05
ALDH3A1	Aldehyde dehydrogenase, dimeric NADP-preferring OS=Homo sapiens	38.35	127.95	3.3363755	1.13E-05

	GN=ALDH3A1 PE=1 SV=3				
KYNU	Kynureninase OS=Homo sapiens GN=KYNU PE=1 SV=1	42.15	137.5	3.262159	1.63E-05
UCHL1	Ubiquitin carboxyl-terminal hydrolase isozyme L1 OS=Homo sapiens GN=UCHL1 PE=1 SV=2	42.3	136.1	3.2174941	2.03E-05
S100P	Protein S100-P OS=Homo sapiens GN=S100P PE=1 SV=2	42.4	134.9	3.1816038	2.43E-05
HIST1H1D	Histone H1.3 OS=Homo sapiens GN=HIST1H1D PE=1 SV=2	46.6	145	3.111588	3.46E-05
AKR1B1	Aldose reductase OS=Homo sapiens GN=AKR1B1 PE=1 SV=3	42.7	131.05	3.0690867	4.28E-05
YBEY	Putative ribonuclease OS=Homo sapiens GN=YBEY PE=1 SV=2	43.35	128.4	2.9619377	7.37E-05
CA12	Carbonic anhydrase 12 OS=Homo sapiens GN=CA12 PE=1 SV=1	44.5	130	2.9213483	9.06E-05
PLSCR1	Phospholipid scramblase 1 OS=Homo sapiens GN=PLSCR1 PE=1 SV=1	50.45	145.25	2.8790882	0.000112375
ANXA4	Annexin (Fragment) OS=Homo sapiens GN=ANXA4 PE=2 SV=1	46.95	134.15	2.857295	0.000125609
HLA-B	MHC class I antigen (Fragment) OS=Homo sapiens GN=HLA-B PE=3 SV=1	50.45	143.75	2.8493558	0.000130813
ASB9	Ankyrin repeat and SOCS box protein 9 OS=Homo sapiens GN=ASB9 PE=1 SV=1	46.75	132.35	2.831016	0.000143682
AKR1C3	Aldo-keto reductase family 1 member C3 OS=Homo sapiens GN=AKR1C3 PE=1 SV=1	45.25	127.4	2.8154696	0.00015559
AGR2	AGR2 OS=Homo sapiens GN=AGR2 PE=4 SV=1	48.85	137	2.8045036	0.000164585
PTGFRN	Prostaglandin F2 receptor	48.35	134.8	2.788004	0.0001791

	negative regulator OS=Homo sapiens GN=PTGFRN PE=1 SV=2			1	19
HLA-C	MHC class I antigen (Fragment) OS=Homo sapiens GN=HLA-C PE=3 SV=1	41.65	116	2.785114	0.000181795
ALDH3A2	Fatty aldehyde dehydrogenase OS=Homo sapiens GN=ALDH3A2 PE=1 SV=1	44.9	124.25	2.7672606	0.000199246
SLC23A2	Solute carrier family 23 (Nucleobase transporters), member 2, isoform CRA_a OS=Homo sapiens GN=SLC23A2 PE=4 SV=1	48.35	132.65	2.7435367	0.000225084
NQO1	NAD(P)H dehydrogenase [quinone] 1 OS=Homo sapiens GN=NQO1 PE=1 SV=1	47.7	129.2	2.7085954	0.000269432
S100A6	Protein S100-A6 OS=Homo sapiens GN=S100A6 PE=1 SV=1	48.5	131.1	2.7030928	0.000277179
SQOR	Sulfide:quinone oxidoreductase, mitochondrial OS=Homo sapiens GN=SQOR PE=1 SV=1	47.1	127.15	2.6995754	0.000282249
SELENOI	Ethanolaminephosphotransferase 1 OS=Homo sapiens GN=SELENOI PE=1 SV=3	49.2	131.95	2.6819106	0.00030916
C15orf48	Chromosome 15 open reading frame 48, isoform CRA_a OS=Homo sapiens GN=C15orf48 PE=4 SV=1	52.85	140.8	2.6641438	0.000338839
PON3	Serum paraoxonase/lactonase 3 OS=Homo sapiens GN=PON3 PE=1 SV=3	48.05	127.05	2.6441207	0.000375748
SPDEF	SAM pointed domain-containing Ets transcription factor OS=Homo sapiens GN=SPDEF PE=1 SV=1	40.45	106.85	2.6415328	0.000380805
COX2	Cytochrome c oxidase	45.1	118.55	2.628603	0.0004071

	subunit 2 OS=Homo sapiens GN=COX2 PE=3 SV=1			1	18
UGDH	UDP-glucose 6-dehydrogenase OS=Homo sapiens GN=UGDH PE=1 SV=1	47.95	125.95	2.626694 5	0.0004111 55
SULT1A4	Sulfotransferase OS=Homo sapiens GN=SULT1A4 PE=1 SV=1	52.45	137.4	2.619637 8	0.0004264 33
AKR1C1	Aldo-keto reductase family 1 member C1 (Fragment) OS=Homo sapiens GN=AKR1C1 PE=1 SV=1	42.4	110.05	2.595518 9	0.0004831 03
HDHD3	Haloacid dehalogenase-like hydrolase domain containing 3, isoform CRA_a OS=Homo sapiens GN=HDHD3 PE=4 SV=1	47	121.95	2.594680 9	0.0004852 03
SHMT1	Serine hydroxymethyltransferase, cytosolic OS=Homo sapiens GN=SHMT1 PE=1 SV=1	52.25	134.7	2.577990 4	0.0005289 95
CREB1	cAMP responsive element binding protein 1 OS=Homo sapiens GN=CREB1 PE=2 SV=1	49.45	126.3	2.554095	0.0005987 13
AKR1C2	Aldo-keto reductase family 1 member C2 OS=Homo sapiens GN=AKR1C2 PE=1 SV=3	46.4	118.45	2.552801 7	0.0006027 4
HEXB	Beta-hexosaminidase subunit beta OS=Homo sapiens GN=HEXB PE=1 SV=3	49.25	125.55	2.549238 6	0.0006139 75
S100A3	Protein S100-A3 OS=Homo sapiens GN=S100A3 PE=1 SV=1	50.9	127.35	2.501964 6	0.0007846 17
LXN	Latexin OS=Homo sapiens GN=LXN PE=1 SV=2	49.05	121.8	2.483180 4	0.0008650 01
LYST	Lysosomal-trafficking regulator OS=Homo sapiens GN=LYST PE=1	60.3	149.7	2.482587 1	0.0008676 71

	SV=3				
CEACAM6	Carcinoembryonic antigen-related cell adhesion molecule 6 OS=Homo sapiens GN=CEACAM6 PE=1 SV=3	51.15	126.65	2.4760508	0.000897635
TNFAIP2	Tumor necrosis factor alpha-induced protein 2 OS=Homo sapiens GN=TNFAIP2 PE=2 SV=2	56.75	138.95	2.4484581	0.00103602
POF1B	Protein POF1B OS=Homo sapiens GN=POF1B PE=1 SV=3	56.4	137.4	2.4361702	0.00110436
MGST1	Microsomal glutathione S-transferase 1 OS=Homo sapiens GN=MGST1 PE=1 SV=1	52.6	124.9	2.3745247	0.00152185
CENPV	Centromere protein V OS=Homo sapiens GN=CENPV PE=1 SV=1	52.25	123.85	2.3703349	0.0015554
ME1	NADP-dependent malic enzyme OS=Homo sapiens GN=ME1 PE=1 SV=1	47.95	113.65	2.3701773	0.00155667
GPRC5C	G-protein-coupled receptor family C group 5 member C OS=Homo sapiens GN=GPRC5C PE=1 SV=1	50.65	119.9	2.3672261	0.00158076
LDHD	Probable D-lactate dehydrogenase, mitochondrial OS=Homo sapiens GN=LDHD PE=1 SV=1	48	113.6	2.3666667	0.00158537
TXN	Thioredoxin OS=Homo sapiens GN=TXN PE=2 SV=1	56.55	133.7	2.3642794	0.00160519
GALM	Aldose 1-epimerase OS=Homo sapiens GN=GALM PE=1 SV=1	51.95	122.6	2.3599615	0.00164167
CXCL5	C-X-C motif chemokine 5 OS=Homo sapiens GN=CXCL5 PE=1 SV=1	63.75	150.35	2.3584314	0.0016548
PBEF1	Pre-B-cell colony enhancing factor 1, isoform	58.5	137.55	2.3512821	0.00171754

	CRA_a OS=Homo sapiens GN=PBEF1 PE=4 SV=1				
HEL107	Epididymis luminal protein 107 OS=Homo sapiens GN=HEL107 PE=2 SV=1	53.8	126.25	2.3466543	0.00175941
RNF13	E3 ubiquitin-protein ligase RNF13 OS=Homo sapiens GN=RNF13 PE=1 SV=1	58.6	137.1	2.3395904	0.00182531
CBR1	Carbonyl reductase [NADPH] 1 OS=Homo sapiens GN=CBR1 PE=1 SV=3	51.25	119.5	2.3317073	0.00190177
MARCKS	Myristoylated alanine-rich C-kinase substrate OS=Homo sapiens GN=MARCKS PE=1 SV=4	60.7	141.2	2.3261944	0.00195714
CEACAM1	Carcinoembryonic antigen-related cell adhesion molecule 1 OS=Homo sapiens GN=CEACAM1 PE=1 SV=2	54.3	124.75	2.2974217	0.00227337
PTGR1	Prostaglandin reductase 1 OS=Homo sapiens GN=PTGR1 PE=1 SV=2	52.75	120.3	2.2805687	0.00248183
LCN2	Neutrophil gelatinase-associated lipocalin OS=Homo sapiens GN=LCN2 PE=1 SV=1	61.95	141.05	2.2768362	0.00253052
IGF2BP1	Insulin-like growth factor 2 mRNA-binding protein 1 OS=Homo sapiens GN=IGF2BP1 PE=1 SV=2	56.25	128.05	2.2764444	0.00253569
SARCOSIN	Sarcomeric muscle protein (Fragment) OS=Homo sapiens GN=SARCOSIN PE=2 SV=1	49.8	113.1	2.2710843	0.00260743
ADD2	Beta-adducin OS=Homo sapiens GN=ADD2 PE=1 SV=3	44.7	101.45	2.2695749	0.002628
MYOF	Myoferlin OS=Homo sapiens GN=MYOF PE=1 SV=1	58.7	133.05	2.2666099	0.00266887
SDCBP	Syntenin-1 OS=Homo sapiens GN=SDCBP PE=1	59.8	135.4	2.264214	0.00270236

	SV=1				
PON2	Paraoxonase 2, isoform CRA_a OS=Homo sapiens GN=PON2 PE=1 SV=1	56.95	127.8	2.2440737	0.00300101
EPHX1	Epoxide hydrolase OS=Homo sapiens GN=EPHX1 PE=2 SV=1	52.25	117.05	2.2401914	0.00306226
PIR	Pirin OS=Homo sapiens GN=PIR PE=1 SV=1	50.45	112.85	2.2368682	0.00311568
FEZ2	Fasciculation and elongation protein zeta-2 (Fragment) OS=Homo sapiens GN=FEZ2 PE=1 SV=1	48.6	108.55	2.2335391	0.00317012
TMEM106B	TMEM106B protein (Fragment) OS=Homo sapiens GN=TMEM106B PE=2 SV=1	56.8	125.95	2.2174296	0.00344727
FAM213A	Redox-regulatory protein FAM213A OS=Homo sapiens GN=FAM213A PE=1 SV=3	58.45	129.45	2.2147134	0.00349633
NCSTN	Nicastrin OS=Homo sapiens GN=NCSTN PE=1 SV=2	57.7	127.6	2.2114385	0.0035564
CTSS	Cathepsin S OS=Homo sapiens GN=CTSS PE=1 SV=3	61.2	135.25	2.2099673	0.00358372
TSPAN15	Tetraspanin-15 OS=Homo sapiens GN=TSPAN15 PE=1 SV=1	58.4	128.6	2.2020548	0.00373429
SRC	Proto-oncogene tyrosine-protein kinase Src OS=Homo sapiens GN=SRC PE=1 SV=3	57.15	125.25	2.191601	0.00394291
AHR	Aryl hydrocarbon receptor, isoform CRA_a OS=Homo sapiens GN=AHR PE=4 SV=1	57.9	125.3	2.164076	0.0045494
CAST	Calpastatin (Fragment) OS=Homo sapiens GN=CAST PE=1 SV=1	57.75	124.45	2.1549784	0.0047696
ICAM1	Intercellular adhesion molecule 1 OS=Homo	61.75	133	2.1538462	0.00479773

	sapiens GN=ICAM1 PE=1 SV=2				
SLC16A3	Solute carrier family 16 (Monocarboxylic acid transporters), member 3, isoform CRA_a OS=Homo sapiens GN=SLC16A3 PE=4 SV=1	60.3	129.5	2.1475954	0.0049560 7
ADIG	Adipogenin (Fragment) OS=Homo sapiens GN=ADIG PE=4 SV=1	56.55	121.4	2.1467728	0.0049772 8
ALDH2	Aldehyde dehydrogenase, mitochondrial OS=Homo sapiens GN=ALDH2 PE=1 SV=2	55.3	118.35	2.1401447	0.0051515 9
C19orf70	MICOS complex subunit MIC13 OS=Homo sapiens GN=C19orf70 PE=1 SV=1	58.25	124.4	2.1356223	0.0052739 8
HLA-A	MHC class I antigen (Fragment) OS=Homo sapiens GN=HLA-A PE=3 SV=1	62.4	132.9	2.1298077	0.0054356
NRAP	Nebulin-related-anchoring protein OS=Homo sapiens GN=NRAP PE=1 SV=1	65.1	138.45	2.1267281	0.0055231 8
MRPL51	39S ribosomal protein L51, mitochondrial OS=Homo sapiens GN=MRPL51 PE=1 SV=1	63.75	135.5	2.1254902	0.0055587 8
DPP3	Dipeptidyl peptidase 3 OS=Homo sapiens GN=DPP3 PE=1 SV=2	58.55	124.15	2.1204099	0.0057072 8
FDFT1	Squalene synthase OS=Homo sapiens GN=FDFT1 PE=1 SV=1	61	129.15	2.1172131	0.0058027 4
RBM47	RNA-binding protein 47 OS=Homo sapiens GN=RBM47 PE=1 SV=2	55.2	116.65	2.1132246	0.0059240 5
TNFSF13B	Tumor necrosis factor ligand superfamily member 13B OS=Homo sapiens GN=TNFSF13B PE=1 SV=1	61.35	128.95	2.1018745	0.0062832 4
EVPL	EVPL protein OS=Homo sapiens GN=EVPL PE=2	56.15	117.55	2.0934996	0.0065620 5

	SV=1				
KRT8	Keratin, type II cytoskeletal 8 OS=Homo sapiens GN=KRT8 PE=1 SV=7	58	121.05	2.087069	0.00678442
TARBP1	Probable methyltransferase TARBP1 OS=Homo sapiens GN=TARBP1 PE=1 SV=1	63.35	132.15	2.08603	0.00682105
ARL8A	ADP-ribosylation factor-like protein 8A OS=Homo sapiens GN=ARL8A PE=1 SV=1	58.1	121.05	2.0834768	0.00691188
CELF2	CUGBP Elav-like family member 2 OS=Homo sapiens GN=CELF2 PE=1 SV=1	59.5	123.85	2.0815126	0.00698257
RPL14	RPL14 protein OS=Homo sapiens GN=RPL14 PE=1 SV=1	59.5	123.65	2.0781513	0.00710522
GPI	Glucose-6-phosphate isomerase (Fragment) OS=Homo sapiens GN=GPI PE=1 SV=1	61	126.75	2.0778689	0.00711561
TBC1D8	TBC1 domain family member 8 OS=Homo sapiens GN=TBC1D8 PE=1 SV=1	63.85	131.85	2.0649961	0.00760606
HEL-S-45	Epididymis secretory protein Li 45 OS=Homo sapiens GN=HEL-S-45 PE=2 SV=1	61.45	126.75	2.0626526	0.00769887
EPB41L1	Band 4.1-like protein 1 OS=Homo sapiens GN=EPB41L1 PE=1 SV=2	59.4	122.35	2.0597643	0.00781481
PLS3	Plastin-3 OS=Homo sapiens GN=PLS3 PE=1 SV=4	61	124.9	2.047541	0.00832491
ADSSL1	Adenylosuccinate synthetase isozyme 1 OS=Homo sapiens GN=ADSSL1 PE=1 SV=1	52.05	106.3	2.0422671	0.00855505
RAB27B	Ras-related protein Rab-27B OS=Homo sapiens GN=RAB27B PE=1 SV=4	64.9	132.4	2.0400616	0.00865313
CEACAM7	Carcinoembryonic antigen-	60.4	121.5	2.011589	0.0100238

	related cell adhesion molecule 7 OS=Homo sapiens GN=CEACAM7 PE=1 SV=1			4	
CDIPT	CDP-diacylglycerol--inositol 3-phosphatidyltransferase OS=Homo sapiens GN=CDIPT PE=1 SV=1	59.65	119.85	2.0092205	0.0101471
ACTR10	Actin-related protein 10 (Fragment) OS=Homo sapiens GN=ACTR10 PE=1 SV=1	61.7	123.65	2.0040519	0.0104213
LMBRD1	Probable lysosomal cobalamin transporter OS=Homo sapiens GN=LMBRD1 PE=1 SV=1	59.45	119	2.0016821	0.0105494
HEL-S-265	Epididymis secretory protein Li 265 OS=Homo sapiens GN=HEL-S-265 PE=2 SV=1	150.45	75.6	0.5024925	0.00413926
PKP2	Plakophilin-2 OS=Homo sapiens GN=PKP2 PE=1 SV=2	144.65	72.55	0.5015555	0.00403594
SMARCA4	Transcription activator BRG1 OS=Homo sapiens GN=SMARCA4 PE=1 SV=1	150.35	75.3	0.5008314	0.00395757
TK1	Thymidine kinase OS=Homo sapiens GN=TK1 PE=1 SV=1	143.25	71.2	0.4970332	0.00356702
MCM5	DNA helicase OS=Homo sapiens GN=MCM5 PE=2 SV=1	148.6	73.85	0.4969717	0.00356098
TSPYL1	Testis-specific Y-encoded-like protein 1 OS=Homo sapiens GN=TSPYL1 PE=1 SV=3	142.6	70.8	0.4964937	0.00351426
MAGED2	Melanoma antigen family D, 2, isoform CRA_a OS=Homo sapiens GN=MAGED2 PE=4 SV=1	147.7	73.3	0.4962762	0.00349317
DDX21	Nucleolar RNA helicase 2 OS=Homo sapiens GN=DDX21 PE=1 SV=5	146	72.3	0.4952055	0.00339091

A2M	Alpha-2-macroglobulin OS=Homo sapiens GN=A2M PE=1 SV=3	146.5	72.2	0.4928328	0.0031732
OXCT1	Succinyl-CoA:3-ketoacid coenzyme A transferase 1, mitochondrial OS=Homo sapiens GN=OXCT1 PE=1 SV=1	148.55	72.85	0.4904073	0.00296289
LIG1	DNA ligase 1 OS=Homo sapiens GN=LIG1 PE=1 SV=1	144.05	70.5	0.4894134	0.00288017
RCN3	Reticulocalbin-3 OS=Homo sapiens GN=RCN3 PE=1 SV=1	147.35	72.1	0.4893112	0.00287177
FBXO30	F-box only protein 30 OS=Homo sapiens GN=FBXO30 PE=1 SV=3	156.05	76.35	0.4892663	0.00286809
EPB41L2	Band 4.1-like protein 2 OS=Homo sapiens GN=EPB41L2 PE=1 SV=1	148.3	72.5	0.4888739	0.00283608
MAP3K4	Mitogen-activated protein kinase kinase kinase 4 OS=Homo sapiens GN=MAP3K4 PE=1 SV=2	138.05	67.3	0.4875045	0.00272673
MT2A	Metallothionein-2 OS=Homo sapiens GN=MT2A PE=1 SV=1	156	75.45	0.4836538	0.00243808
FHL3	Four and a half LIM domains protein 3 OS=Homo sapiens GN=FHL3 PE=1 SV=4	148.8	71.95	0.4835349	0.0024296
KHDRBS3	KH domain-containing, RNA-binding, signal transduction-associated protein 3 OS=Homo sapiens GN=KHDRBS3 PE=1 SV=1	152	73.3	0.4822368	0.00233857
HEL-S-69	Epididymis secretory protein Li 69 OS=Homo sapiens GN=HEL-S-69 PE=1 SV=1	155.05	74.65	0.4814576	0.00228533
MTHFD2	Bifunctional methylenetetrahydrofolate dehydrogenase/cyclohydro	141.3	67.95	0.4808917	0.00224731

	lase, mitochondrial OS=Homo sapiens GN=MTHFD2 PE=1 SV=2				
CTPS1	CTP synthase 1 OS=Homo sapiens GN=CTPS1 PE=1 SV=2	150.6	71.95	0.4777556	0.00204618
HEL-S-277	DDAH2 OS=Homo sapiens GN=HEL-S-277 PE=1 SV=1	150.75	72	0.4776119	0.00203734
TPM2b	Beta tropomyosin isoform OS=Homo sapiens GN=TPM2b PE=2 SV=1	157.2	74.6	0.4745547	0.00185684
RANBP1	RAN binding protein 1, isoform CRA_g OS=Homo sapiens GN=RANBP1 PE=2 SV=1	154.9	73.4	0.4738541	0.00181744
CRMP1	Dihydropyrimidinase-related protein 1 OS=Homo sapiens GN=CRMP1 PE=1 SV=1	144.9	68.65	0.473775	0.00181304
HIST1H1C	Histone H1.2 OS=Homo sapiens GN=HIST1H1C PE=1 SV=2	160.85	76	0.4724899	0.00174277
hCG_28765	HCG28765, isoform CRA_b OS=Homo sapiens GN=hCG_28765 PE=4 SV=1	152.9	71.95	0.470569	0.00164207
AHNAK	Neuroblast differentiation-associated protein AHNAK OS=Homo sapiens GN=AHNAK PE=1 SV=2	155.75	73.25	0.470305	0.00162862
HLA-B	MHC class I antigen OS=Homo sapiens GN=HLA-B PE=3 SV=1	158.4	73.8	0.4659091	0.00141815
SDC4-ROS1_S4;R32	Tyrosine-protein kinase receptor OS=Homo sapiens GN=SDC4-ROS1_S4;R32 PE=2 SV=1	144.4	66	0.4570637	0.00106419
CLN6	Ceroid-lipofuscinosis neuronal 6 late infantile variant isoform 1 OS=Homo sapiens GN=CLN6 PE=2 SV=1	152.1	69.5	0.4569362	0.0010597
TUBA2	Tubulin alpha chain OS=Homo sapiens	157.25	71.35	0.4537361	0.000952265

	GN=TUBA2 PE=2 SV=1				
SH3BP1	SH3 domain-binding protein 1 OS=Homo sapiens GN=SH3BP1 PE=1 SV=3	152.7	69.15	0.4528487	0.000924188
NASP	Nuclear autoantigenic sperm protein OS=Homo sapiens GN=NASP PE=1 SV=2	154.75	70	0.4523425	0.000908492
MAGEA1	Melanoma-associated antigen 1 OS=Homo sapiens GN=MAGEA1 PE=1 SV=1	163.85	74.05	0.4519377	0.00089611
IMPDH2	Inosine-5'-monophosphate dehydrogenase 2 OS=Homo sapiens GN=IMPDH2 PE=1 SV=2	154.7	69.9	0.4518423	0.00089321
DUT	Deoxyuridine 5'-triphosphate nucleotidohydrolase, mitochondrial OS=Homo sapiens GN=DUT PE=1 SV=4	153.5	69.25	0.4511401	0.000872131
TOP2A	DNA topoisomerase 2-alpha OS=Homo sapiens GN=TOP2A PE=1 SV=3	149.35	67	0.4486106	0.000799713
EPHA2	Ephrin type-A receptor 2 OS=Homo sapiens GN=EPHA2 PE=1 SV=2	155.55	69.7	0.4480874	0.0007854
PAGE2B	Putative G antigen family E member 3 OS=Homo sapiens GN=PAGE2B PE=3 SV=1	154.55	69.15	0.447428	0.000767676
TAGLN	Transgelin OS=Homo sapiens GN=TAGLN PE=2 SV=1	159.2	71.05	0.446294	0.000738003
UBE2S	Ubiquitin-conjugating enzyme E2 S OS=Homo sapiens GN=UBE2S PE=1 SV=2	154.9	68.5	0.4422208	0.000639447
RRM2B	Ribonucleoside-diphosphate reductase subunit M2 B (Fragment)	156.7	69.25	0.4419272	0.000632808

	OS=Homo sapiens GN=RRM2B PE=1 SV=1				
PTGFRN	PTGFRN protein (Fragment) OS=Homo sapiens GN=PTGFRN PE=2 SV=1	161.9	70.6	0.4360716	0.000512288
PBK	Lymphokine-activated killer T-cell-originated protein kinase OS=Homo sapiens GN=PBK PE=1 SV=3	157.5	68.6	0.4355556	0.000502693
FSCN1	Fascin OS=Homo sapiens GN=FSCN1 PE=1 SV=3	160.7	69.4	0.4318606	0.000438453
NUDT1	7,8-dihydro-8-oxoguanine triphosphatase OS=Homo sapiens GN=NUDT1 PE=1 SV=3	157.7	67.75	0.4296132	0.000402984
PODXL	Podocalyxin OS=Homo sapiens GN=PODXL PE=2 SV=1	167.6	71.65	0.427506	0.000372028
TMSB10	Thymosin beta-10 OS=Homo sapiens GN=TMSB10 PE=1 SV=2	162.4	68.75	0.4233374	0.000316854
RPL22L1	60S ribosomal protein L22-like 1 (Fragment) OS=Homo sapiens GN=RPL22L1 PE=1 SV=1	165.4	69.95	0.4229141	0.000311674
UAP1	UDP-N-acetylhexosamine pyrophosphorylase OS=Homo sapiens GN=UAP1 PE=1 SV=3	162.85	68.6	0.4212465	0.000291983
PDLIM4	PDZ and LIM domain protein 4 OS=Homo sapiens GN=PDLIM4 PE=1 SV=2	159.4	66.75	0.4187578	0.000264623
HMGB2	High mobility group protein B2 OS=Homo sapiens GN=HMGB2 PE=1 SV=2	160.6	67	0.4171856	0.00024852
TPM2	Tropomyosin beta chain OS=Homo sapiens GN=TPM2 PE=1 SV=1	162.4	67.05	0.4128695	0.000208657
KRT75	Keratin, type II cytoskeletal 75 OS=Homo sapiens GN=KRT75 PE=1 SV=2	158.6	65.35	0.4120429	0.000201701
CXorf67	Uncharacterized protein CXorf67 OS=Homo sapiens	163.8	67.25	0.4105617	0.000189745

	GN=CXorf67 PE=1 SV=1				
SLC1A5	Neutral amino acid transporter B(0) OS=Homo sapiens GN=SLC1A5 PE=1 SV=2	159.1	64	0.4022627	0.000133609
HLA-A	MHC class I antigen (Fragment) OS=Homo sapiens GN=HLA-A PE=3 SV=1	157.95	63.35	0.4010763	0.000126923
LCP1	Lymphocyte cytosolic protein 1 (L-plastin), isoform CRA_a OS=Homo sapiens GN=LCP1 PE=4 SV=1	155.15	61.9	0.3989687	0.000115774
PROCR	Endothelial protein C receptor OS=Homo sapiens GN=PROCR PE=1 SV=1	158.6	63.1	0.3978562	0.000110246
hCG_2014768	HCG2014768, isoform CRA_a OS=Homo sapiens GN=hCG_2014768 PE=4 SV=1	174.75	69.4	0.3971388	0.000106806
RBP1	Retinol binding protein 1, cellular OS=Homo sapiens GN=RBP1 PE=1 SV=1	166.45	65.65	0.3944127	9.46E-05
GNL3	Guanine nucleotide-binding protein-like 3 OS=Homo sapiens GN=GNL3 PE=1 SV=2	144.95	57	0.393239	8.97E-05
LGALS1	Galectin-1 OS=Homo sapiens GN=LGALS1 PE=1 SV=2	170.45	66.45	0.3898504	7.69E-05
hCG_23833	HCG23833, isoform CRA_b OS=Homo sapiens GN=hCG_23833 PE=4 SV=1	163.35	63.6	0.389348	7.51E-05
IL8	Multifunctional fusion protein OS=Homo sapiens GN=IL8 PE=3 SV=1	175.05	67.8	0.3873179	6.84E-05
QPRT	Nicotinate-nucleotide pyrophosphorylase [carboxylating] OS=Homo sapiens GN=QPRT PE=1 SV=3	139.6	54	0.3868195	6.68E-05

KCTD14	BTB/POZ domain-containing protein KCTD14 OS=Homo sapiens GN=KCTD14 PE=1 SV=2	165.9	64.1	0.386377 3	6.55E-05
STMN1	Stathmin OS=Homo sapiens GN=STMN1 PE=2 SV=1	170.5	65.45	0.383871	5.82E-05
CRYAB	Alpha-crystallin B chain OS=Homo sapiens GN=CRYAB PE=1 SV=2	157.7	60.15	0.381420 4	5.18E-05
H1F0	Histone H1.0 OS=Homo sapiens GN=H1F0 PE=1 SV=3	171.5	65.3	0.380758	5.02E-05
PHGDH	D-3-phosphoglycerate dehydrogenase OS=Homo sapiens GN=PHGDH PE=1 SV=4	166.9	62.75	0.375973 6	3.97E-05
FGF1	Fibroblast growth factor 1 OS=Homo sapiens GN=FGF1 PE=1 SV=1	156.75	58.5	0.373205 7	3.46E-05
CKB	Creatine kinase brain isoform 2 (Fragment) OS=Homo sapiens GN=CKB PE=2 SV=1	169.35	60.5	0.357248 3	1.51E-05
YA61	Drug-sensitive protein 1 OS=Homo sapiens GN=YA61 PE=2 SV=1	171.85	61.2	0.356124 5	1.42E-05
EEF1D	Elongation factor 1-delta (Fragment) OS=Homo sapiens GN=EEF1D PE=1 SV=1	172.35	59	0.342326 7	6.47E-06
MMP3	Stromelysin-1 OS=Homo sapiens GN=MMP3 PE=1 SV=2	173.45	59.25	0.341597	6.20E-06
TNC	TNC variant protein (Fragment) OS=Homo sapiens GN=TNC variant protein PE=2 SV=1	171	57.55	0.336549 7	4.58E-06
BCAT1	Branched-chain-amino-acid aminotransferase OS=Homo sapiens GN=BCAT1 PE=3 SV=1	177.45	59.5	0.335305 7	4.24E-06
DHRS2	Dehydrogenase/reductase SDR family member 2,	176.7	58.85	0.333050 4	3.69E-06

	mitochondrial OS=Homo sapiens GN=DHRS2 PE=1 SV=4				
SERPINB11	Serpin B11 OS=Homo sapiens GN=SERPINB11 PE=1 SV=2	168	55.75	0.3318452	3.43E-06
NDN	Necdin OS=Homo sapiens GN=NDN PE=2 SV=1	187.85	61.55	0.327655	2.63E-06
S100A2	Protein S100-A2 OS=Homo sapiens GN=S100A2 PE=1 SV=2	185	60.15	0.3251351	2.23E-06
TUBB2B	Tubulin beta-2B chain OS=Homo sapiens GN=TUBB2B PE=1 SV=1	170.2	53.45	0.3140423	1.06E-06
FLNC	Filamin-C OS=Homo sapiens GN=FLNC PE=1 SV=3	180.7	56.45	0.3123962	9.49E-07
GSTZ1	Maleylacetoacetate isomerase OS=Homo sapiens GN=GSTZ1 PE=1 SV=3	184.55	57.25	0.310214	8.14E-07
PHF15	PHD finger protein 15, isoform CRA_a OS=Homo sapiens GN=PHF15 PE=2 SV=1	183.65	55.2	0.3005717	4.04E-07
MTAP	S-methyl-5'-thioadenosine phosphorylase OS=Homo sapiens GN=MTAP PE=4 SV=1	182.05	53.45	0.2936007	2.37E-07
CCNB1	G2/mitotic-specific cyclin-B1 OS=Homo sapiens GN=CCNB1 PE=1 SV=1	185.1	52	0.2809292	8.48E-08
IGF2BP2	Insulin-like growth factor 2 mRNA-binding protein 2 OS=Homo sapiens GN=IGF2BP2 PE=1 SV=1	188.95	52.25	0.2765282	5.82E-08
KRT17	Keratin, type I cytoskeletal 17 OS=Homo sapiens GN=KRT17 PE=1 SV=2	188.8	51.5	0.2727754	4.19E-08
CSTA	Cystatin-A OS=Homo sapiens GN=CSTA PE=1 SV=1	191.55	49.25	0.257113	9.71E-09
PTX3	Pentraxin-related protein PTX3 OS=Homo sapiens	192.75	48.3	0.2505837	5.04E-09

	GN=PTX3 PE=1 SV=3				
HIST1H1A	Histone H1.1 OS=Homo sapiens GN=HIST1H1A PE=1 SV=3	203.1	46.95	0.2311669	5.97E-10
PLEKHH2	Pleckstrin homology domain-containing family H member 2 OS=Homo sapiens GN=PLEKHH2 PE=1 SV=2	203.25	44.4	0.2184502	1.25E-10
MTAP	Purine nucleoside phosphorylase OS=Homo sapiens GN=MTAP PE=2 SV=1	203.35	42.9	0.2109663	4.63E-11
S100A2	Protein S100-A2 OS=Homo sapiens GN=S100A2 PE=1 SV=3	209.9	36.55	0.1741305	1.32E-13
HEL-S-2a	Epididymis secretory sperm binding protein Li 2a OS=Homo sapiens GN=HEL-S-2a PE=2 SV=1	229.1	34.45	0.150371	9.59E-16

Table S2. shRNA or siRNA sequences used throughout this study.

Gene		Sequences
**pro-SFTPB**	shRNA#1	5′- CAGACTGACTCAAACGGCATCTGTA -3′
		5′- TACAGATGCCGTTTGAGTCAGTCTG -3′
	shRNA#2	5′- CACAGGATCTCTCCGAGCAGCAATT -3′
		5′- AATTGCTGCTCGGAGAGATCCTGTG -3′
	shRNA#3	5′- TGCAAGCAATTTGTGGAGCAGCACA -3′
		5′- TGTGCTGCTCCACAAATTGCTTGCA -3′
**Scramble**	shRNA	5′- TTCTCCGAACGTGTCACGTAA -3′
		5′- TTACGTGACACGTTCGGAGAA -3′
**ADRM1**	siRNA#1	5′- GACGACUCGCUUAUUCACU -3′
		5′- AGUGAAUAAGCGAGUCGUC -3′
	siRNA#2	5′- GCUUCCCUACUUGCCAUCU -3′
		5′- AGAUGGCAAGUAGGGAAGC -3′
	siRNA#3	5′- CAUGCAGCUCAUCGGACCA -3′
		5′- UGGUCCGAUGAGCUGCAUG -3′
**siUCH37**	siRNA#1	5′- CAUUUAGGCGAGACAUUAU -3′
		5′- AUAAUGUCUCGCCUAAAUG -3′
	siRNA#2	5′- GCUCAUUAAAGGAUUCGGU -3′
		5′- ACCGAAUCCUUUAAUGAGC -3′
	siRNA#3	5′- CCAGCAGUUAAUACCACUA -3′
		5′- UAGUGGUAUUAACUGCUGG -3′
**siPGK1**	siRNA#1	5′- CCAAGUCGGUAGUCCUUAU -3′
		5′- AUAAGGACUACCGACUUGG -3′
	siRNA#2	5′- GUCGUUAUGAGAGUCGACU -3′
		5′- AGUCGACUCUCAUAACGAC -3′
	siRNA#3	5′- CCAAGAUUGUCAAAGACCU -3′
		5′- AGGUCUUUGACAAUCUUGG -3′
**Negative control**	siRNA	5′- UUCUCCGAACGUGUCACGU -3′
		5′- ACGUGACACGUUCGGAGAA -3′

Table S3 Characteristics of patients with early-stage lung adenocarcinoma (For IHC)

**Variable**	Total (n=160)	**SFTPB expression levels**		***p***
		Normal (n=72)	Low (n=88)	
**Gender**				0.78
Female	78	36	42	
Male	82	36	46	
**Age**				0.94
60≤	85	38	47	
60>	75	34	41	
**Stage**				0.13
I	145	68	77	
II	15	4	11	
**Smoking status**				0.46
NO	104	49	55	
Yes	56	23	33	
**Recurrence status**				0.002
Recurrence	90	31	59	
Non-recurrence	70	41	29	
**PGK1**				0.004
High	89	31	58	
Low	71	41	30	
**p-AKT**				0.002
High	95	33	62	
Low	65	39	26	

Table S4 Characteristics of patients with early-stage lung adenocarcinoma (for serum)

**Variable**	Total (n=160)	**SFTPB expression levels**		***p***
		Normal (n=113)	Low (n=24)	
**Gender**				0.17
Female	45	40	5	
Male	92	73	19	
**Age**				0.27
60≤	66	52	14	
60>	71	61	10	
**Stage**				0.27
I	93	79	14	
II	44	34	10	
**Recurrence status**				0.00
Recurrence	57	39	18	
Non-recurrence	80	74	6	
